# Supplementary material for: High-Performance Sample Substrate of Gold Nanoparticle Multilayers for Surface-Assisted Laser Desorption/Ionization Mass Spectrometry
Source: Nanomaterials (Basel). 2019 Jul 27;9(8):1078. doi: 10.3390/nano9081078 (PMC6723548; doi:10.3390/nano9081078)
Supplement: Supplementary file 1 [file nanomaterials-09-01078-s001.pdf]

nanomaterials

Article

High-Performance Sample Substrate of Gold Nanoparticle Multilayers for Surface-Assisted Laser Desorption/Ionization Mass Spectrometry

Yen-Chen Liu [1,†], Yi-Hsuan Chang [1,†], Yun-Ho Lin [2,3,†], Chien-Chung Liou [4] and Tsung-Rong Kuo [1,5,6,*]

[1] Graduate Institute of Nanomedicine and Medical Engineering, College of Biomedical Engineering, Taipei Medical University, Taipei 11031, Taiwan
[2] Division of Oral Pathology, Department of Dentistry, Taipei Medical University Hospital, Taipei 11031, Taiwan
[3] School of Dentistry, College of Oral Medicine, Taipei Medical University, Taipei 11031, Taiwan
[4] Department of Chemistry, Tunghai University, Taichung 40704, Taiwan
[5] International Ph.D. Program in Biomedical Engineering, College of Biomedical Engineering, Taipei Medical University, Taipei 11031, Taiwan
[6] Research Center of Biomedical Device, Taipei Medical University, Taipei 11031, Taiwan
* Correspondence: trkuo@tmu.edu.tw
† These authors contributed equally to this work.

Received: 4 July 2019; Accepted: 23 July 2019; Published: date

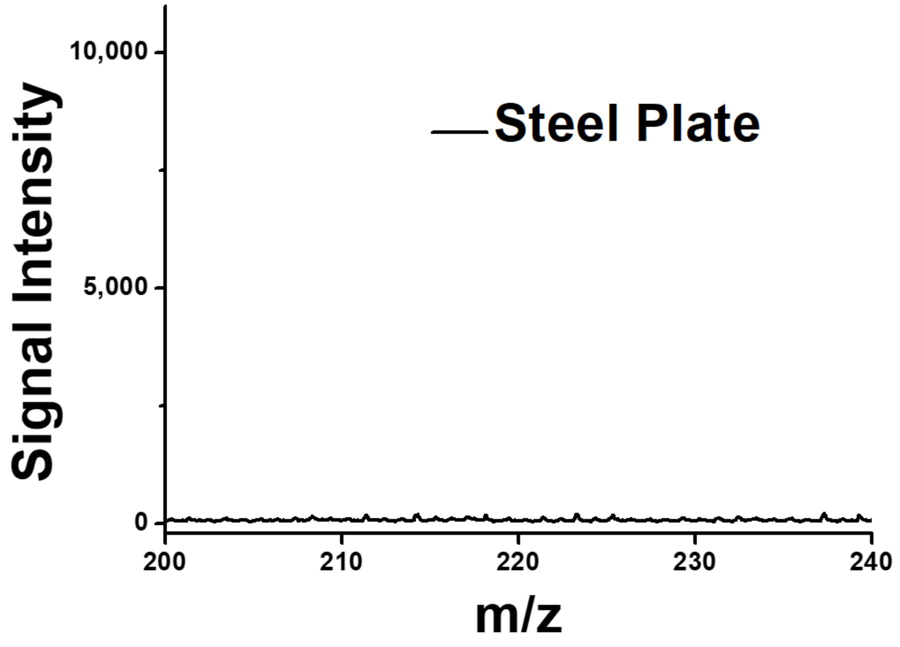

Figure S1. Mass spectra of glucose using steel plate without coating in SALDI-MS.

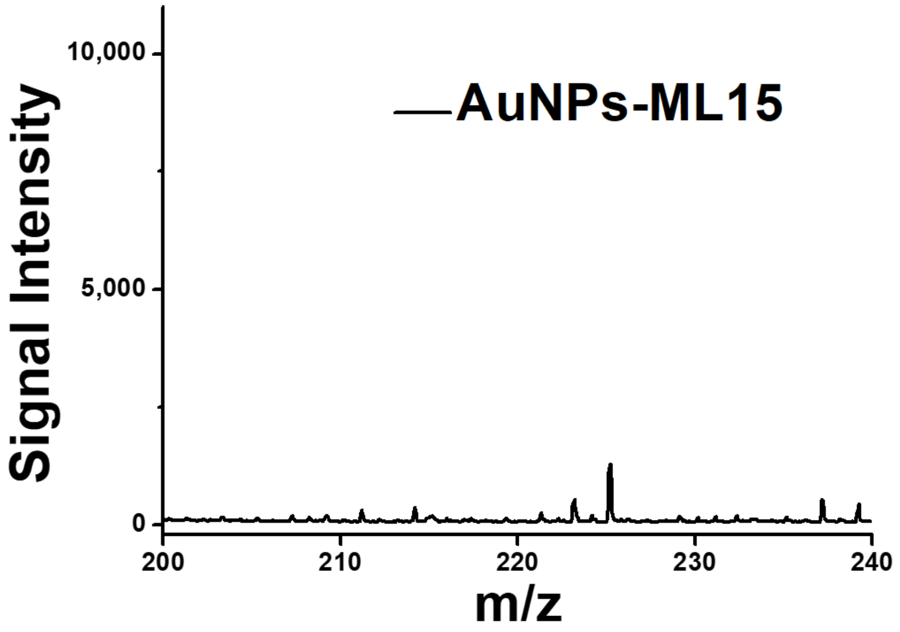

Figure S2. The background signals of AuNPs-ML15 sample substrate in SALDI-MS.
